# Comparative plasma and urine metabolomics analysis of juvenile and adult canines

**DOI:** 10.3389/fvets.2022.1037327

**Published:** 2023-01-09

**Authors:** Taibo Wu, Yun Chen, Mingzi Yang, Shuang Wang, Xiaoming Wang, Manli Hu, Xu Cheng, Juan Wan, Yufeng Hu, Yi Ding, Xin Zhang, Mingxing Ding, Zhengming He, Hongliang Li, Xiao-Jing Zhang

**Affiliations:** ^1^School of Basic Medical Science, Wuhan University, Wuhan, China; ^2^Institute of Model Animal, Wuhan University, Wuhan, China; ^3^Clinical Trial Centers, Huanggang Central Hospital, Huanggang, China; ^4^College of Veterinary Medicine, Huazhong Agricultural University, Wuhan, China; ^5^School of Basic Medical Science, Key Laboratory of Cardiovascular Disease Prevention and Control, Ministry of Education, Gannan Medical University, Ganzhou, China; ^6^Gannan Innovation and Translational Medicine Research Institute, Gannan Medical University, Ganzhou, China; ^7^Institute of Laboratory Animal Resources, National Institutes for Food and Drug Control, Beijing, China

**Keywords:** metabolomics, growth, plasma, urine, canines

## Abstract

**Background and aims:**

The metabolomic profile of a biofluid can be affected by age, and thus provides detailed information about the metabolic alterations in biological processes and reflects the in trinsic rule regulating the growth and developmental processes.

**Methods:**

To systemically investigate the characteristics of multiple metabolic profiles associated with canine growth, we analyzed the metabolomics in the plasma and urine samples from 15 young and 15 adult beagle dogs via UHPLC-Q-TOFMS-based metabolomics. Blood routine and serum biochemical analyses were also performed on fasting blood samples.

**Results:**

The metabolomics results showed remarkable differences in metabolite fingerprints both in plasma and urine between the young and adult groups. The most obvious age-related metabolite alterations include decreased serumlevels of oxoglutaric acid and essential amino acids and derivatives but increased levels of urine levels of O-acetylserine. These changes primarily involved in amino acid metabolism and bile secretion pathways. We also found that the levels of glutamine were consistently higher in both serum and urine of adults, while N-acetylhistamine and uracil concentrations were much lower in the adult group compared to younger ones.

**Conclusion:**

Our study provides a whole metabolic profile of serum and urine characteristics of young and adult canines, identifying several metabolites that were significantly associated with age change, which provides theoretical support for the nutrition-related research and age-related homeostasis maintenance in dogs.

## 1. Introduction

Metabolic homeostasis is a highly dynamic status during the development from juvenile to adult, accompanied by robust changes in physiological metabolites, proteins, cells, and organs. The establishment of metabolic homeostasis is thus closely correlated with and controls nutritional demands, pathophysiologic responses, and disease susceptibilities (1, 2), including atopic diseases, hematological malignancies, autoimmune diseases, diabetes, and cardiovascular diseases (3, 4). Therefore, understanding the growth and age-related molecular mechanisms and metabolic changes is essential for health management and disease prevention.

Metabolomics allows comprehensive detection and analysis of endogenous small molecule compounds in the biological samples, including the substrates, intermediates, and products of metabolic reactions. Since metabolites are the final downstream products in the biological system and contain significant information reflecting age, sex, lifestyle, dietary intake, and disease settings, metabolomics is closer to phenotype than transcriptomics or proteomics (5). Metabolomics has become a powerful tool for unraveling metabolic details and screening important biomarkers of diseases, such as aging, cancer, diabetes, obesity, and cardiovascular disease (6–8).

Currently, most metabonomic studies have demonstrated specific metabonomic characteristics affected by age. For example, Pann et al. found that the plasma metabolomics of mice had high variability at different ages. B6J mice had high levels of acylcarnitines at week 6 and high levels of phenylalanine, ornithine, and tyrosine at week 14 (9). In addition, the histidine/histamine pathway (10), tryptophan, purine, amino acid and nicotinamide metabolism (11), glycine, serine and threonine metabolism, glyoxylic acid, and dicarboxylic acid metabolism pathways in mice are significantly correlated with age (12–15). In a urine metabonomic analysis of healthy children, the researchers found that these significantly age-related metabolites in infancy were amino acid and fatty acid metabolic pathways and carbohydrate metabolism (16). Through metabonomic analysis of plasma and urine samples of young and elderly people, a variety of age-related metabolites were found, such as glutamine, aspartate, glutamate, sphingolipids, trimethylamine N-oxide, etc., mainly involving amino acid and fatty acid metabolic pathways (17, 18). Understanding the metabolic process of the body at different ages and clarifying the role of key metabolites in health, growth and development as well as aging will provide important information for the formulation of nutrition requirements for different stages and anti-aging preventive measures. Furthermore, metabolomics can be further used to investigate the relationship between diseases and age. It has been identified that blood metabolites and metabolic pathways for PD (Parkinson's Disease) are significantly associated with age, and developed serine as a blood biomarker for the early diagnosis of Parkinson's Disease (PD) (19).

Previous studies have provided strong evidence that metabolomics analysis is an important tool for investigating metabolic changes associated with aging. But comprehensive information about the plasma and urine metabolic networks of dogs and how they are affected by age and other factors is not yet fully understood. With the increasing numbers of pets and evolving of the pet food and medical industry, researchers have focused on the research of healthier pet foods or targeted therapeutic drugs for certain diseases. For example, grain-free pet foods supplemented with methionine, taurine, or methyl donors and methyl receivers can increase the concentration of homocysteine, methionine, or taurine in the plasma after a meal. Supplementing these nutrients can meet the canine's body's demand for amino acids and help to treat diseases that cause metabolic or oxidative stress (20). Dietary calcium fructoborate supplementation can reduce the degree of joint pain in osteoarthritis dogs (21). Therefore, understanding the information about the metabolic alterations in different growth stages could further reveal the mechanisms by which age influences body metabolism and disease risk, and identify the biomarkers with potential clinical utilities, which is vital for formulating nutritional needs at different stages and preventing age-related chronic diseases. In addition, dogs have become a new model of aging biology and age-related diseases. With the growth of age, dogs experience functional decline similar to that of humans, and develop many age-related diseases, including cancer, metabolic syndrome, and neurodegeneration. Therefore, scientific findings on dogs may be helpful to the research on human biology and aging (22, 23).

In our study, an LC-MS-based untargeted metabolomics platform was used to depict the whole alterations of blood and urine metabolomic profiles in dogs of different ages. To reveal the metabolic pathways related to age, we conducted KEGG analysis and correlation analyses on the differential metabolites in the two groups, and finally determined the major age-related metabolites and age-related changes in metabolic pathways, such as amino acid metabolism and bile secretion.

## 2. Materials and methods

### 2.1. Animal

Healthy juvenile beagle puppies (*n* = 15, age range = 3–6 months old; mean ± SD = 4 ± 1 months) and healthy adult beagle dogs (*n* = 15, age range = 12–70 months; mean ± SD = 41± 22 months) were obtained from Hubei yizhicheng Biotechnology Co., Ltd. All of the dogs were fed in single cages under the same and stable environmental conditions with temperature at 25°C and relative humidity at 75%. The study protocols were approved by the Ethical Committee of Gannan Medical College.

### 2.2. Collection and preparation of blood and urine

Blood samples were collected *via* blood collection needle from the dog's forearm vein into the BD Vacutainer tubes (BD-Vacutainer; Franklin Lakes, NJ) on the same morning under fasting conditions. EDTA whole blood was analyzed the complete blood count using an VetScan HM5 automatic blood cell analyzer (Abaxis, USA). Blood samples in the heparin anticoagulant tube were centrifuged at 3,000 rpm for 10 min at 4°C to separate plasma. One plasma sample was sent to WellAnimal Test (Wuhan) Co., Ltd. for biochemical detection using an automatic biochemical analyzer (Hitachi 3110; Hitachi, Tokyo, Japan). The remaining plasma was stored at −80 °C freezer until metabolomic analysis.

For urine collection and storage, dogs were anesthetized with a mixture of Zoletil (50 mg/kg) and xylazine (3 mg/kg). Urine was collected by bladder puncture under the direction of a Doppler ultrasonic apparatus. Urine samples were centrifuged at 3,000 rpm for 10 min at 4°C to remove sediments and then stored at −80°C freezers until metabolomic analysis.

### 2.3. Plasma and urine metabolomic analysis

Non-targeted metabolomic analysis of plasma and urine samples was performed using ultra-high performance liquid chromatography (UHPLC; Agilent 1290 Infinity LC System) equipped with Triple TOF 6600 mass spectrometer (AB SCIEX, USA). Chromatographic separation was implemented on Waters, ACQUITY UPLC BEH Amide 1.7 μm, 2.1 × 100 mm column for both positive and negative models. To guarantee the quality of non-targeted metabolomic analysis, 6 QC samples were inserted during metabonomic analysis process. They were slowly thawed at 4 °C, and vortex mixed in pre-cooled extraction buffer (methanol/acetonitrile/water,2:2:1, v/v). Then, mixtures were sonicated for 30 min at 4 °C, settle for 10 min at −20°C, and centrifuged at 14,000 × g for 20 min at 4°C. The supernatant was collected, vacuum-dried, and resolubilized in 100 μL acetonitrile-water solution (1:1, v/v). The supernatant was separated on UHPLC. The Column temperature was 25 °C, the injection volume was 2 μL and the flow rate was 0.5 mL/min. Then, the samples were analyzed by Triple TOF 6600 mass spectrometers. The ESI parameters were as follows: ion source gas 1 (Gas 1), 60; ion source gas 2 (Gas 2), 60; curtain gas (CUR), 30 psi; source temperature, 600°C; and ion spray voltage floating (ISVF) ± 5,500 V (positive and negative modes). The mass spectrometry data were collected in the Information-dependent acquisition (IDA) mode. The declustering potential (DP) was ±60 V (positive and negative modes); the collision energy (CE) was 35 V with ± 15 eV; Exclude isotopes within 4 Da, and 10 candidate ions were monitored per cycle.

The MS raw data (wiff files) were converted to mzXML format using ProteoWizard, and then peak alignment, retention time correction, and peak area extraction were performed using the R package XCMS program. Based on the self-built database [in-house database (Shanghai Applied Protein Technology)], compound identification of metabolites was performed by matching with the information of the retention time of metabolite, accuracy m/z value (<10 ppm), fragmentation analysis, and the identification results were strictly checked and confirmed manually.

### 2.4. Multidimensional statistical analysis

Orthogonal partial least-square discriminant analysis (OPLS-DA) were used for multidimensional statistical analysis. OPLS-DA is a supervised multivariate statistical method and was applied to establish the correlation between metabolites expression and sample as well as to evaluate the variation (R2Y) and predictive ability of the model (Q2) by 7-fold cross-validation. The variable importance in projection (VIP) values in the OPLS-DA model (VIP > 1) and *p*-value (*P* < 0.05) were used as the criteria for further screening of significant differential metabolites.

### 2.5. Analysis of pathway and differential metabolites

To identify the most important metabolic pathways during growth and development, the enrichment analysis was performed with KEGG pathway analysis (Kyoto Encyclopedia of Genes and Genomes, http://www.kegg.jp/). Correlations between phenotypic and main differential metabolites in plasma, and differential metabolites in plasma and urine were carried out by Spearman's rank correlation.

### 2.6. Statistics

Data were presented as mean ± standard, and 20.0 SPSS (IBM Corporation, Chicago, USA) was used to analyze whether the data conformed to normal distribution. The two-tailed Student's *t*-test was applied to assess the differences between two groups conforming to normal distribution. If not correspondent with normal distribution, the differences between the two groups was compared using the Mann-Whitney *U* nonparametric test. The level of significance is shown by asterisks: ^*^P < 0.05, ^**^P < 0.01, ^***^P < 0.001.

## 3. Results

### 3.1. Age-related changes in the number of blood cells

**Figure 1:**
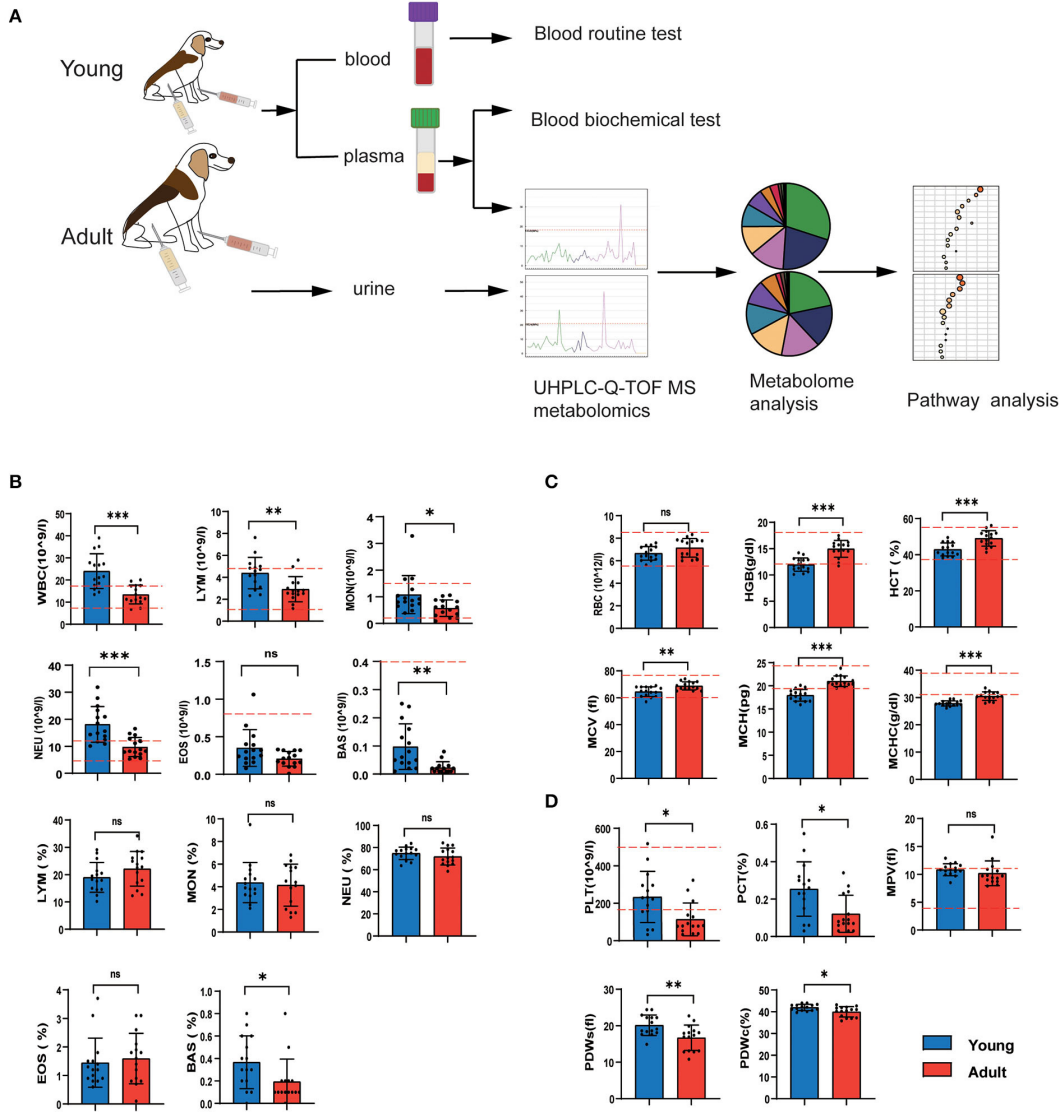
Age-related changes in the number of blood cells. **(A)** Study flow diagram of tests of physiological and biochemical indexes, UHPLC-Q-TOF/MS detection, metabolite analysis and pathway analysis. **(B–D)** Routine blood texts of young and adult groups. The white blood cell-derived indexes **(B)**, red blood cell-related indexes **(C)**, platelet-derived indices **(D)**. Horizontal red dotted lines represent the normal range for each measured value. *n* = 15 dogs per group. Data are represented as mean and standard deviation. Adult group vs. young group: *P < 0.05, **P < 0.01, ***P < 0.001, and ns indicates not significantly different.

To investigate the phenotypic variations during canine development, we first collected blood samples that were subjected to routine blood tests, biochemistry examinations and non-targeted metabolomics (Figure 1A). The results showed that the levels of most routine blood indexes fell within the normal range of established standards for dogs according to the analyzer's software (Figures 1B–D). We found that young dogs had higher levels of leukocyte-related parameters compared with adult dogs, including white blood cell (WBC) counts, lymphocyte (LYM) counts, monocytes (MON) counts, eosinophils (EOS) counts, and neutrophil (NEU) counts, and eosinophil (EOS) (Figure 1B). While the red blood cell-related parameters except red blood cell count (RBC) were much higher in adults as compared to young dogs (Figure 1C). The levels of platelet count were conversely lower in the adult group compared with the younger ones (Figure 1D).

### 3.2. Liver and heart function biomarkers were significantly separated between adult and young canines

**Figure 2:**
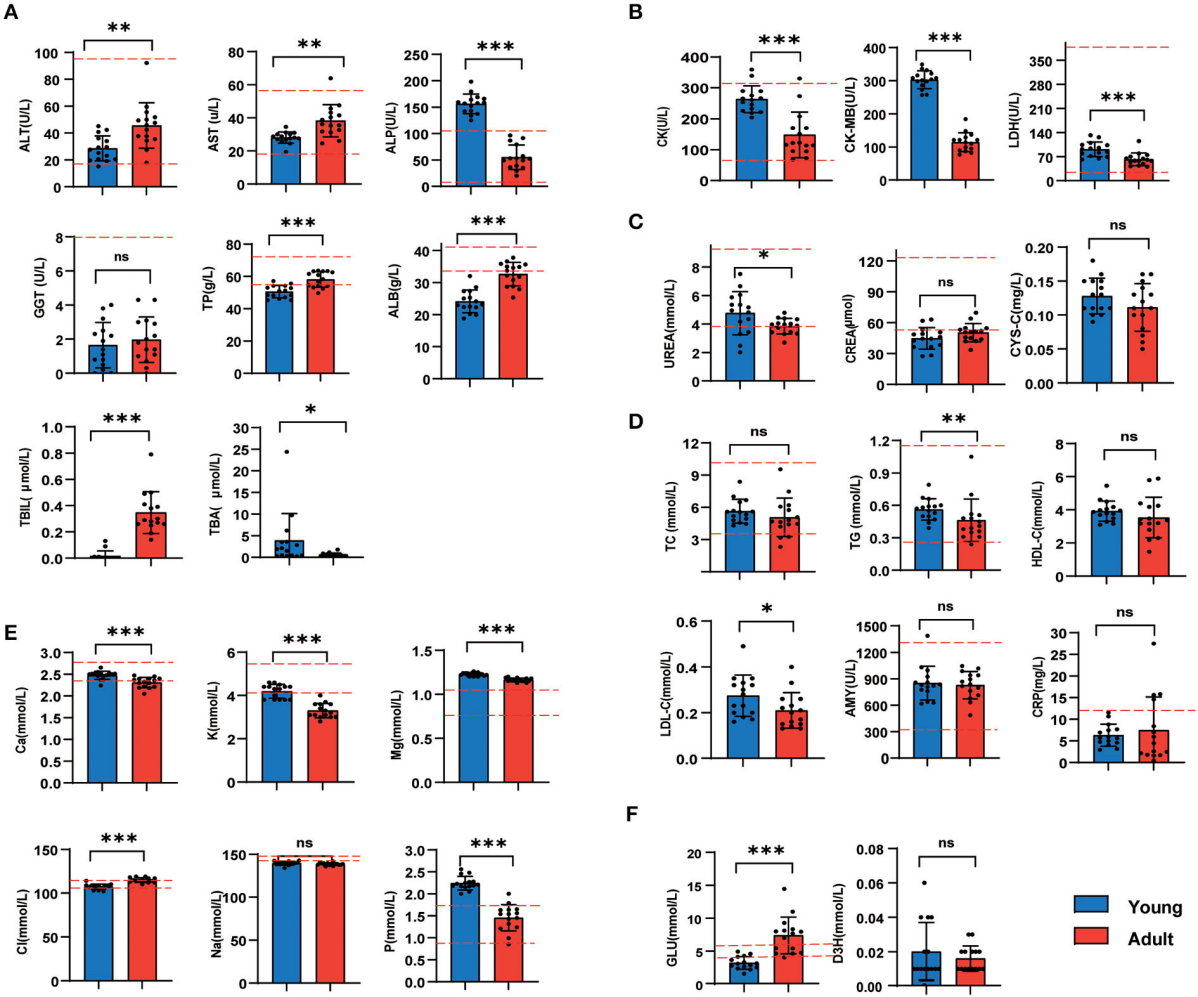
Liver and heart function biomarkers were significantly separated between adult and young canines **(A–E)**. The levels of serum biochemical indicators in young dogs and adult dogs. Measurement of liver function biomarkers **(A)**, heart function biomarkers **(B)**, kidney function indices **(C)**, blood lipids **(D)**, and blood ion concentrations **(E)**. **(F)** Fasting blood glucose and serum β-hydroxybutyrate levels were measured under overnight fasting conditions. Horizontal red dotted lines represent the normal range for each measured value. *n* = 15 dogs per group. Data are represented as mean and standard deviation. Adult group vs. young group: *P < 0.05, **P < 0.01, ***P < 0.001, and ns indicates not significantly different.

As vital biochemical indicators indicating the liver function, the ALT, AST, TP, ALB, and TBIL activities were considerably lower in the young group than those in the adult group (*p* < 0.05, Figure 2A). In contrast, the content of plasma myocardial enzymes (LDH, CK, and CK-MB) was significantly decreased along with the development of beagle dogs (Figure 2B). Furthermore, we observed levels of kidney function indexes and blood lipids were largely similar between the two groups (Figures 2C, D). In terms of plasma ions, the calcium, potassium, magnesium, and phosphorus concentrations were much lower in the adult group (Figure 2E), but the fasting blood glucose level in the adult group was higher than younger ones (Figure 2F).

### 3.3. Plasma metabolomics profiles were largely remodeled during canine growth

**Figure 3:**
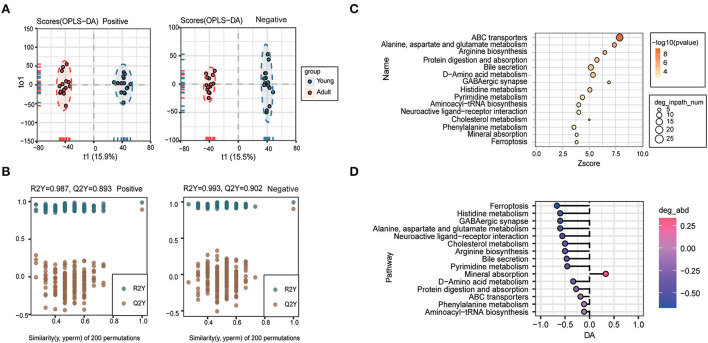
Plasma metabolomics profiles were largely remodeled during canine growth. **(A, B)** Orthogonal partial least squares discriminant analysis (OPLS-DA) score plot **(A)** and validation plot of the OPLS-DA model **(B)** of the young group and the adult group in positive (left) and negative (right) ion modes. **(C, D)** Pathway analysis for plasma differential metabolites between young and adult groups was performed based on the Kyoto Encyclopedia of Genes and Genomes (KEGG) pathway enrichment analysis. The 15 most significantly enriched pathways **(C)** and differential abundance (DA) score of these pathways **(D)**.

To deeply investigate the age-related whole changing spectrums in metabolic phenotypes, plasma samples of young dogs and adult dogs were collected and subjected to untargeted metabolomics analysis. A total of 1,498 metabolites were identified in the plasma. As a reliable multivariate statistical method, OPLS-DA was applied to display the differences in metabolites between samples from the two groups. As shown in Figure 3A, the clustering of the young group was well separated from that of the adult group based on the negative and positive ion modes. The model evaluation parameters (for positive ion mode: R_2_Y = 0.987, Q_2_Y = 0.893; for negative ion mode: R_2_Y = 0.993, Q_2_Y = 0.902) showed that the OPLS-DA model was good in both stability and reliability and can be used for further analysis (Figure 3B).

To identify the age-associated pathway, the KEGG pathway enrichment analysis was carried out according to the differential metabolites (VIP > 1, *P* < 0.05), and the top 15 most significantly enriched pathways were shown in Figure 3C. Among them, differentially-expressed metabolites mainly participated in pathways of ABC transporters, alanine, aspartate and glutamate metabolism, arginine biosynthesis, protein digestion and absorption, bile secretion, and histidine metabolism (Figure 3C). The 15 KEGG pathways were further ranked based on their differential abundance scores. Of note, the most significantly decreased pathways included ferroptosis, histidine metabolism, alanine, aspartate and glutamate metabolism, neuroactive ligand-receptor interaction, cholesterol metabolism, arginine biosynthesis, and bile secretion pathway (Figure 3D).

### 3.4. Amino acid and bile metabolism pathways were compromised in adult canines

**Figure 4:**
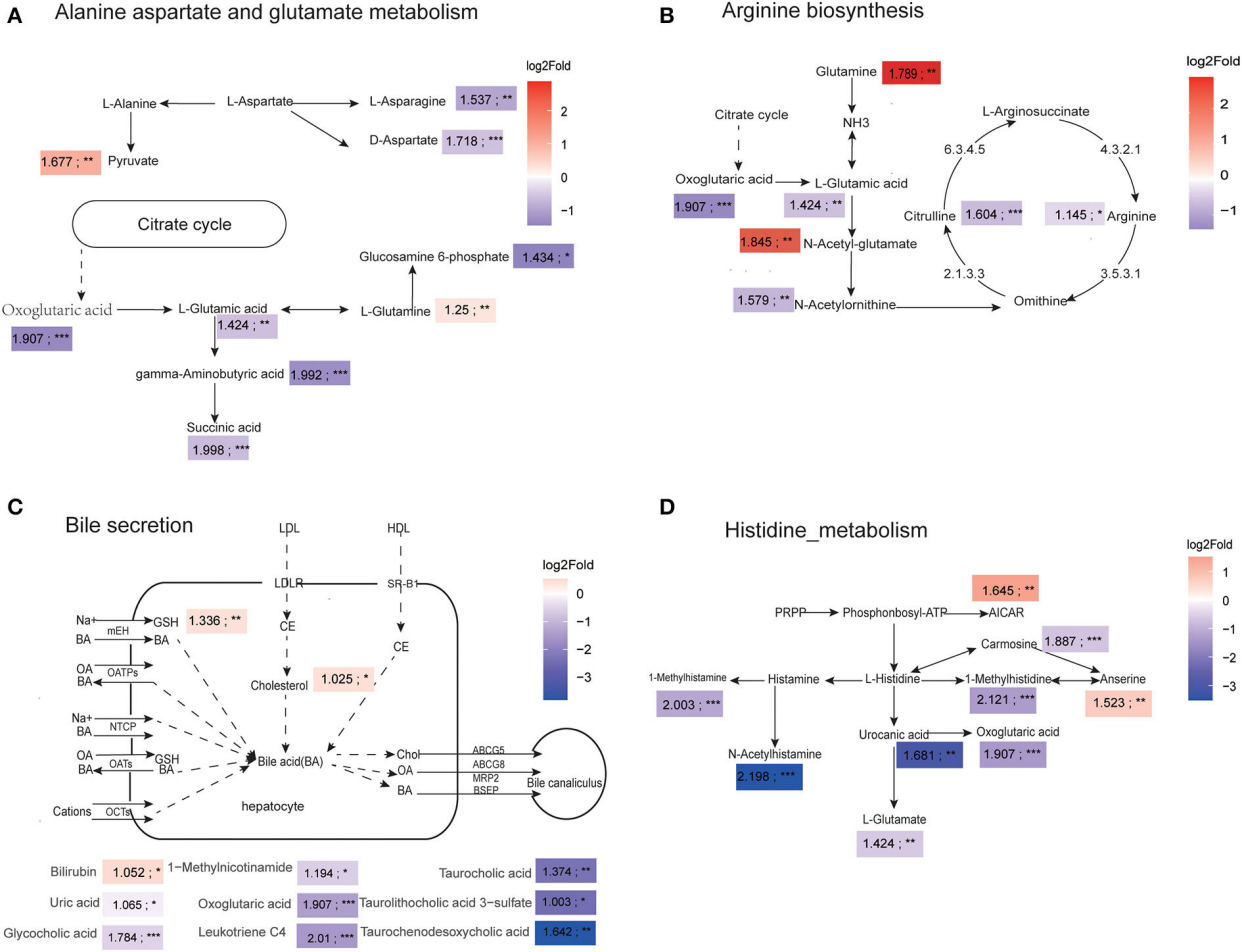
Amino acid and bile metabolism pathways were compromised in adult canines. **(A–D)** The four major metabolic networks showed significantly changed metabolites in alanine aspartate and glutamate metabolism pathway **(A)**, arginine biosynthesis pathway **(B)**, bile secretion pathway **(C)**, and histidine metabolism pathway **(D)**. Color scale represents log2 fold change. Red represents metabolite abundances in adult dogs higher than that in young dogs and purple depicts metabolites with decreased abundance in adult dogs relative to young dogs; The value represents the VIP value of the OPLS-DA model; *P < 0.05, **P < 0.01, ***P < 0.001 vs. young group.

We comprehensively compared the number of differentially changed metabolites and DA scores of each pathway, and selected four mainly altered metabolic pathways for further analysis, including alanine, aspartate and glutamate metabolism, arginine biosynthesis, bile secretion, and histidine metabolism. For the alanine, aspartate, and glutamate metabolism pathways, we found that the level of L-glutamic acid and oxoglutaric acid were significantly decreased in adult dogs, which lead to reductions in gamma-aminobutyric acid and succinic acid content (Figure 4A). The metabolic map of the arginine biosynthesis reveals that the concentrations of L-arginine, citrulline were significantly decreased, which were associated with low L-glutamic acid and N-acetylornithine levels (Figure 4B). As shown in Figure 4C, the adult dogs had higher plasma cholesterol levels and lower plasma bile acids compared to the young dogs. Particularly, glycocholic acid, taurocholic acid, taurolithocholic acid 3-sulfate, and taurochenodesoxycholic acid were significantly decreased in the adult group. Figure 4D showed that the degradation products of histidine were significantly decreased with age, such as 1-methylhistidine, N-acetylhistamine, urocanic acid, and 1-methylhistamine, indicating a decrease in histidine metabolism (Figure 4D).

### 3.5. Plasma metabolite profiles were correlated with age-related physiological index

**Figure 5:**
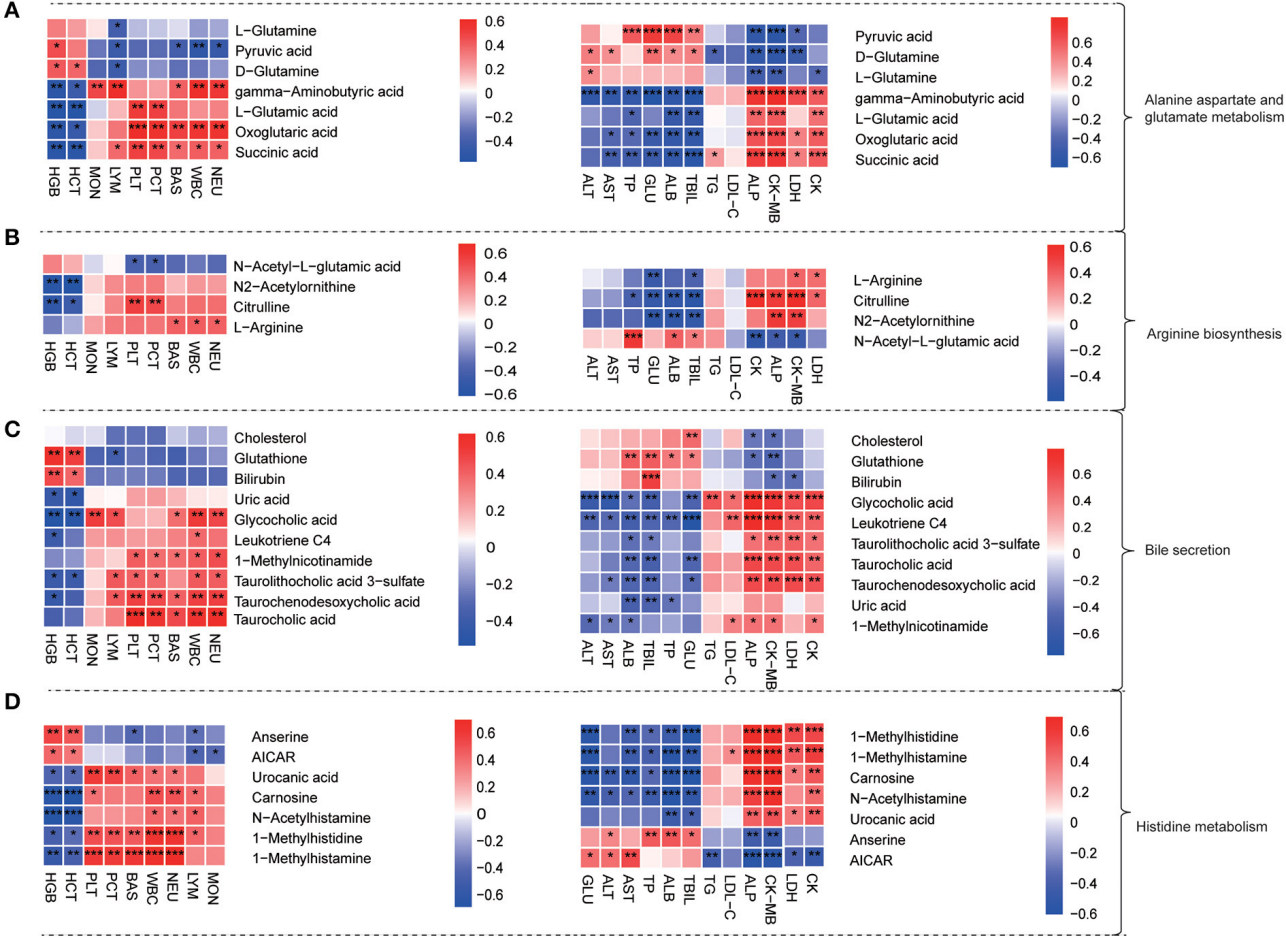
Plasma metabolite profiles were correlated with age-related physiological index. **(A–D)** Heat map of the correlation between physiological indexes (significantly changed blood routine indicators and serum biochemical indicators) with differential plasma metabolites from alanine aspartate and glutamate metabolism pathway **(A)**, arginine biosynthesis pathway **(B)**, bile secretion pathway **(C)**, and histidine metabolism pathway **(D)**; Heat map: red represented positive correlation and blue represented negative correlation. Significance levels *P < 0.05, **P < 0.01, ***P < 0.001.

To further explore the physiological significances of plasma differential metabolites, we analyzed the correlations between the level of dramatically changed metabolites with that of routine blood and biochemical indexes (Figure 5). The results showed that the gamma-aminobutyric acid, oxoglutaric acid, succinic acid, taurocholic acid, 1-methylhistidine, and 1-methylhistamine were positively correlated with WBC, NEU, BAS, PLT, and PCT. The gamma-aminobutyric acid, glycocholic acid, 1-methylhistidine, 1-methylhistamine, and carnosine were positively correlated with CK, CK-MB, LDH, and ALP but negatively correlated with ALT, AST, TBIL, and ALB. As shown in Figure 5D, the differential metabolites in the histidine metabolism pathway were the strongest ones correlated with both blood cell counts and organ heart functional indexes.

### 3.6. Urine metabolite profile undergone significantly change during body growth

**Figure 6:**
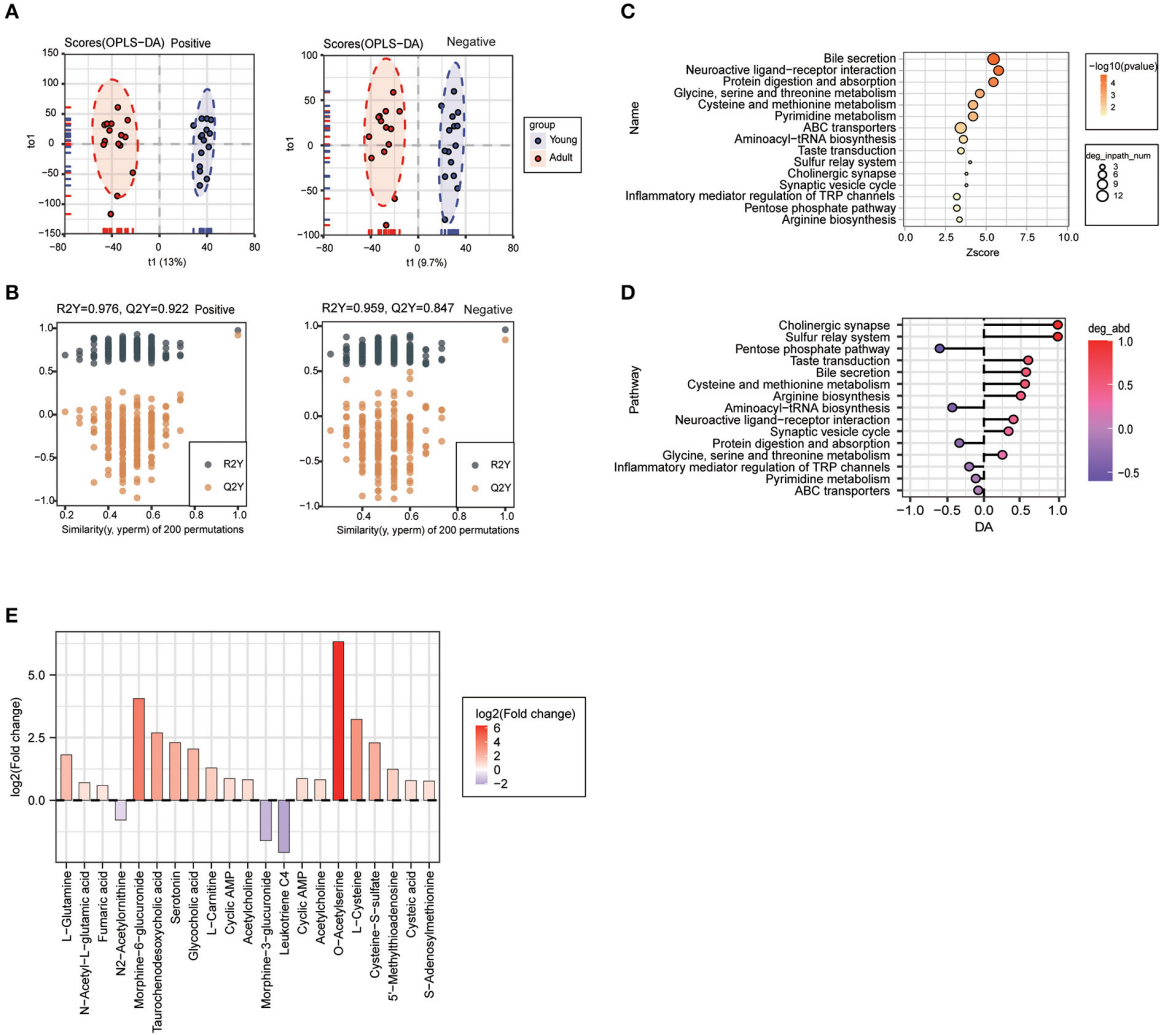
Urine metabolite profile undergone significantly change during body growth. OPLS-DA score plot **(A)** and OPLS-DA validation plots **(B)** urine samples from the young group and adult group. **(C)** KEGG pathway enrichment analysis of differential metabolites in comparison between the two groups, and the top 15 significantly enriched pathways are listed. **(D)** Differential abundance analysis of top 15 KEGG pathways. **(E)** Analysis of the urine differential metabolites in adult dogs compared with young dogs. Red represents increased and purple represented decreased abundance in adult dogs.

Since urine metabolic profile is closely correlated with body development and physiological status, we further carried out urine metabolomics assay using UHPLC-Q-TOF MS in both positive and negative ion scan modes. The OPLS-DA model showed a clear cluster differentiation of the metabolic profiles between the young and adult groups (Figure 6A) with satisfactory evaluation indexes reflecting a high stability of the model (Figure 6B).

A total of 600 differential metabolites were identified and mapped into 32 KEGG pathways, and the 15 most significantly enriched pathways were shown in Figure 6C, mainly including bile secretion, neuroactive ligand-receptor interaction, protein digestion and absorption, glycine, serine and threonine metabolism, and other metabolic processes. Clearly, we noted that cholinergic synapse, bile secretion, sulfur relay system, taste transduction, cysteine and methionine metabolism pathways were significantly upregulated in the adult group, while the pentose phosphate pathway as well as protein digestion and absorption pathways were largely inactivated along with body growth (Figure 6D). Among metabolites involved in those enriched pathways, O-acetylserine, morphine-6-glucuronide, and L-cysteine were identified as the most significantly changed ones in the urine samples from adult animals compared to the younger ones (Figure 6E).

### 3.7. Amino acids and their derivatives were conserved metabolites reflecting development in both plasma and urine

**Figure 7:**
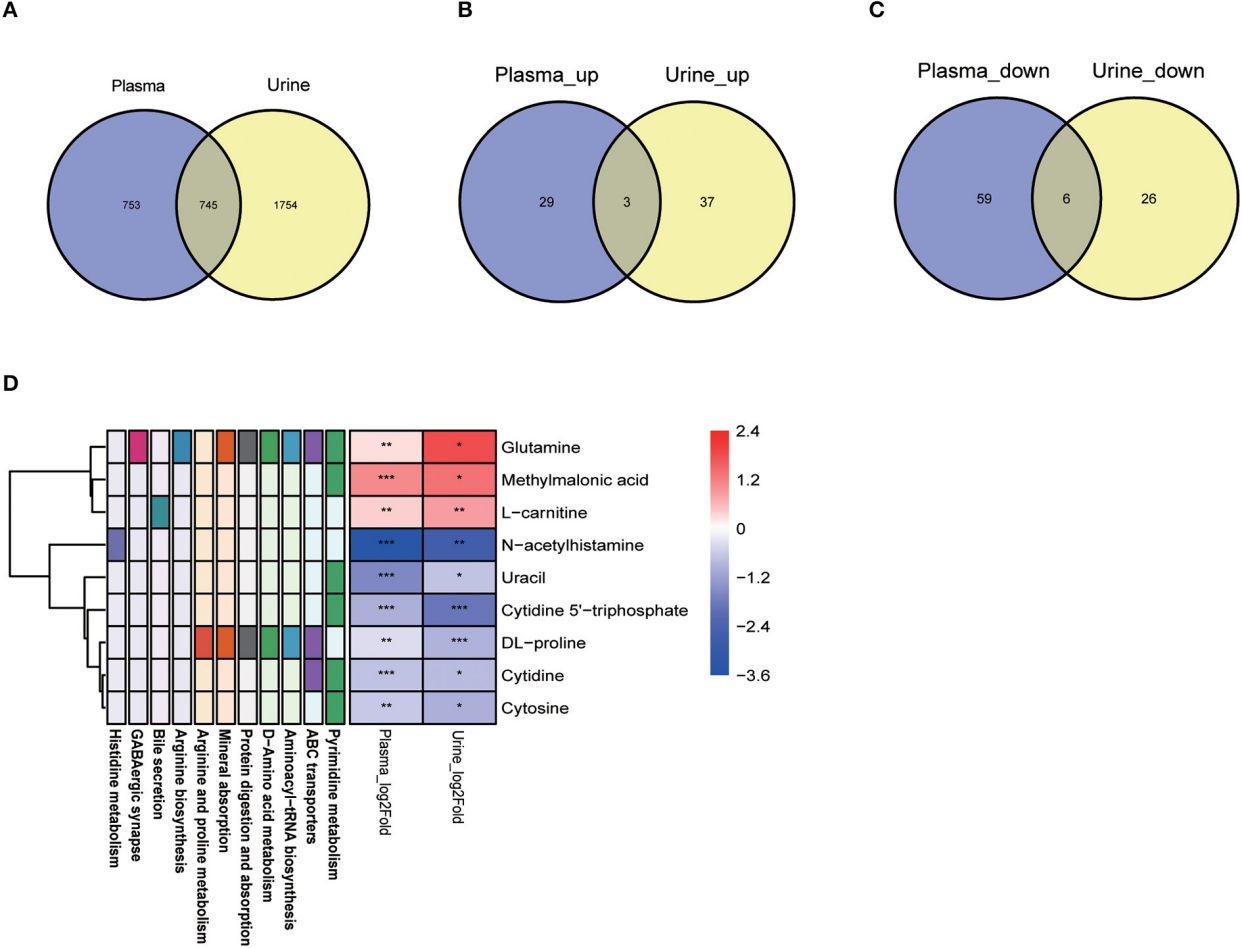
Amino acids and their derivatives were conserved metabolites reflecting development in both plasma and urine. **(A)** Venn diagram showed 1,498 and 2,499 metabolites identified from plasma and urine, respectively; **(B, C)** Only three significantly increased metabolites and six significantly decreased metabolites were observed in both the plasma and the urine of adult dogs. Purple represents the plasma samples and yellow represents the urine samples. **(D)** The nine metabolites derived from **(B, C)** involved in multiple metabolic pathways. For the metabolite lists: red represents increased (VIP > 0) and purple represented decreased abundance (VIP < 0) in adult dogs. For the metabolic pathways: different colors of the box represent the different metabolic pathways involved by the corresponding metabolites. ^*^P < 0.05, ^**^P < 0.01, ^***^P < 0.001 vs. young group.

Our combined metabolomics analysis revealed that 745 metabolites were identified in both the plasma and urine samples (Figure 7A), among which 9 conserved differential metabolites were identified (Figures 7B, C). Notably, glutamine, methylmalonic acid and L-carnitine were all significantly higher in adult body fluid samples than those in young group (Figure 7D). In contrary, the abundance of 6 metabolites, such as N-acetylhistamine and uracil, were much lower in both the plasma and urine of adult ones (Figure 7D). These conserved metabolites were related to 11 KEGG pathways, in particular pyrimidine metabolism and ABC transporters pathways (Figure 7D).

## 4. Discussion

Age is one of the main factors affecting physiology and metabolic changes. In the present study, the age-related phenotypic variations were evidenced by blood routine, serum biochemical tests, and plasma and urine metabolites. In terms of blood routine tests, we observed higher levels of WBC and PLT but lower RBC levels in young dogs than that in adults. The age-associated changes in hematological reported in this study are consistent with those findings in dogs reported in previous studies (24, 25). Brenten T et al. regularly collected the blood of Labrador retriever and miniature schnauzer dogs between weeks 8 and 52 for blood routine and blood biochemical analysis, and found that WBC count decreased over time, RBC, HGB, HCT, MCV, and MCH increased with age (26). Ishii T et al. compared the blood routine data from juvenile beagle dogs (<3 months of age) with dogs (6 months of age) and found that the count of RBC increased and WBC decreased with the growth of beagles (27). RBC maturation and HGB concentration were positively correlated with age (28, 29).

Blood biochemistry is a useful diagnostic tool to assess organ system health. It is necessary to understand the age-related changes in blood chemistry. In this study, AST and ALT activities were significantly higher in the adult group than those in the young group, The effect of age on ALT and AST activity is consistent with prior results. Ishii T et al. analyzed the different of blood chemistry data between the two age classes, and found that the growth of beagle dogs was shown to be associated with increases in AST, ALT, and with decreases in CK (27). ALT is primarily localized in the liver, while AST is expressed in liver, cardiac muscle, and skeletal muscle. All of them are important enzymes in the liver and are involved in the formation of oxalacetic and pyruvic acids respectively. Serum AST and ALT levels increased with age, which is closely related to tissue growth and muscle mass (26). When the metabolism of liver cells or muscle cells in the body is enhanced, the intracellular transaminase enters the serum after cell rupture, resulting in the increase of AST and ALT concentrations in plasma. In addition, the activities of ALT and AST in serum are important indicators to evaluate the extent of hepatic injury (30). Early studies identified that increased AST and ALT levels in aged mice suggest impairment of the liver and metabolic syndrome (31–33). Conversely, higher serum ALP levels were observed in young dogs, which might be due to the production of bone-specific ALP, an ALP isoenzyme, elevating as a result of skeletal growth and increased osteoblastic activity (34, 35). In addition, due to physiological differences between young and adult dogs, some indicators of young dogs were not within the normal range of blood routine or blood biochemical indexes for adult dogs (Figures 1, 2). Therefore, it is very important to identify the changes of age-related physiological indicators and establish reference intervals for blood routine and biochemical indicators in dogs of different ages.

Regarding the metabolomics profiles, several pathways were significantly different between young and adult dogs, mainly including amino acid metabolism (alanine aspartate and glutamate metabolism, arginine biosynthesis, histidine metabolism), bile secretion, and cholesterol metabolism. In particular, the levels of oxoglutaric acid, gamma-aminobutyric acid, L-Glutamic, D-Aspartate and succinic acid were decreased in adult dogs compared to young dogs. These metabolome characteristics of dogs showed high similarity to those in human studies (12). As a key intermediate of the tricarboxylic acid (TCA) cycle, oxoglutaric acid is a significant regulatory metabolite that coordinates nitrogen and carbon metabolism, which has the physiological function of regulating nitrogen metabolism, energy metabolism, maintaining intestinal health, and improving bone (36). The basal diet supplemented with oxoglutaric acid can improve the glutamine metabolism of intestinal cells, enhance intestinal digestion and absorption function, and improve the bone mineral density and performance of piglets (37, 38). In addition, oxoglutaric acid is a biosynthetic precursor of many amino acids such as L-glutamic acid, L-glutamine, succinic acid, and L-arginine acid (39). Glutamic acid is an important neurotransmitter in the central nervous system. It can be catalyzed by glutamic acid decarboxylase (GAD) to produce Gamma-aminobutyric acid (GABA), which is also a well-known neurotransmitter (40, 41). Roalf DR et al. found lower levels of brain Glu in healthy older adults compared to younger adults, leading to age-related declines in behavioral and cognitive function, indicating changes in glutamate as a marker for age-related neurological changes (42).

Blood arginine levels are maintained by food protein, protein turnover, and endogenous synthesis from citrulline through the small intestine-kidney metabolic axis (43). High plasma citrulline concentration in neonates and neonatal pigs was utilized by the kidney for arginine synthesis *via* the intestinal-renal axis (44). The arginine biosynthesis pathway was inhibited in adult dogs, which may due to inhibition of the absorption in the intestine or *de novo* synthesis of arginine (45). Arginine, the precursor for the production of intracellular NO, involved in the regulation of vascular tone and immune regulation, improving cardiovascular function in hypertension and pulmonary hypertension (46). Exogenous supplement of arginine can increase the synthesis and release of NO, and has significant effects on enhancing blood flow, lowering blood pressure and improving arteriosclerosis. L-citrulline is precursor of L-arginine, which more effective and bioavailable because it has a longer circulation time (46). Clinical studies showed that arginine supplementation, in the form of either arginine or citrulline, can improve pulmonary hemodynamics and blood circulation in the cerebral microvasculature, have therapeutic benefits in children with MELAS syndrome (47–49). In addition, arginine is involved in the body's protein composition, and lack of arginine in young animals will lead to delayed production and reduced immunity. It has been found that adding arginine to the feed of piglets can improve immune status and growth performance (50, 51).

Histidine pathway analyses revealed that the plasma levels of 1-methylhistidine, 1-methylhistamine, urocanic acid, and N-acetylhistamine in the young dogs were significantly higher than those in adult dogs, indicating that active histidine metabolism in young dog. Histidine is a dietary essential amino acid with unique roles in the scavenging of reactive oxygen and nitrogen species, erythropoiesis, and the histaminergic system (52, 53). At present, histidine has been used as a nutritional supplement under various conditions. histidine can improve the gastric mucosal damage induced by aspirin in rats (54). Combined use of histidine and vitamin C can reduce free radical induced toxicity, and prevent isoproterenol-induced cardiotoxicity (55). It can also inhibit inflammation and oxidative stress, enhance skin barrier function, and reduce the severity of AD (56). In addition, L-histidine combines with beta-alanine to synthesize the carnosine, which is widely abundant in skeletal muscle, heart, and brain tissues. Carnosine has the function of anti-oxidant, free-radical scavenging and exercise performance-enhancing. The level of carnosine is associated with gender and age, and decreased with age (57, 58). This study found that carnosine levels in dogs showed the same trend.

Compared with younger plasma, adult canines possess lower taurocholic acid and some bile acid abundances but higher cholesterol concentration in adult plasma samples. Bile acid, an important solution for digesting fats, is metabolized from cholesterol in the liver and secreted into the duodenum for digestion and absorption of ingested lipids and fat-soluble vitamins (59). In earlier studies of the human gallstone disease, Kim et al. (60) have found that the liver synthesis of primary bile acid was decreased and the cholesterol secretion was enhanced with age, further increasing the cholesterol saturation of bile, resulting in an increased probability of developing gallstones. Han et al. showed that the decrease in bile acid synthesis and secretion was associated with liver aging by transplanting livers of young rats into old rats (61). Additionally, lowered bile acid secretion was unfavorable for anti-allergic and anticarcinogenic (62). Recent studies indicate that certain probiotic supplementation, such as Bifidobacteria, could reduce cholesterol levels *via* converting cholesterol into the cell membrane of probiotics (59).

Analyzing urine metabolites between young and adult dogs, we found that the bile secretion pathway was the remarkably enriched KEGG pathway. The urine of adult dogs was characterized by the higher content of O-acetylserine, mor-phine-6-glucuronide, and L-cysteine, and the lower content of leukotriene C4. O-acetylserine is a central metabolite of sulfur assimilation, providing the carbon backbone for catalyzing cysteine formation (63). Cysteine can be directly taken off sulfhydryl and amino to generate pyruvate, which can form acetyl-CoA through oxidative decarboxylation, and then be further converted to glutamate (64, 65). Cysteine, as a glutathione precursor, has antioxidant potential. N-acetylcysteine and cysteine-rich whey protein isolate are usually used as cysteine supplements in clinical studies. N-acetylcysteine was shown to increase whole blood glutathione levels and decrease the basal insulin reactivity in AIDS patients and non-diabetic obese patients, respectively (66, 67). Additionally, cysteine-rich undenatured whey protein could significantly improve the skeletal muscle function, decreased plasma levels of the inflammatory cytokine tumor necrosis factor-α (TNF-α), improved immune functions, and increased plasma albumin levels (68).

In conclusion, the present study systematically profiled the phenotypic and metabolic characteristics of young and adult beagle dogs. Our findings showed apparent variations in blood routine and biochemical indexes, and, more importantly, supported a dramatic metabolite remodeling in body fluids during canine growth. In recent years, the addition of functional ingredients in food is becoming a new direction of pet foods research and development to fulfill the specific requirements for the growth and improve the organismal fitness (21, 69). Analyzing the dynamic metabolic process could firmly guide food research and development and disease therapy.

## Data availability statement

The original contributions presented in the study are included in the article/Supplementary material, further inquiries can be directed to the corresponding authors.

## Ethics statement

The animal study was reviewed and approved by the Animal Ethics Committee of the Gannan Medical University.

## Author contributions

JW, X-JZ, YC, and HL designed the research. TW, MY, and SW performed the research and acquired the data. XW, XC, YH, and YD analyzed the data. X-JZ, YC, TW, MY, MH, and XZ wrote the manuscript. All authors were involved in revising the manuscript and approved the final version before submission.

## References

[1] Zhang X Yang Y Su J Zheng X Wang C Chen S. Age-related compositional changes and correlations of gut microbiome, serum metabolome, and immune factor in rats. Geroscience. (2021) 43:709–25. 10.1007/s11357-020-00188-y 32418021 PMC8110635

[2] Barzilai N Huffman DM Muzumdar RH Bartke A. The critical role of metabolic pathways in aging. Diabetes. (2012) 61:1315–22. 10.2337/db11-1300 22618766 PMC3357299

[3] Passarino G De Rango F Montesanto A. Human longevity: genetics or lifestyle? It takes two to tango. Immun Ageing. (2016) 13:12. 10.1186/s12979-016-0066-z 27053941 PMC4822264

[4] Slupsky CM Rankin KN Wagner J Fu H Chang D Weljie AM. Investigations of the effects of gender, diurnal variation, and age in human urinary metabolomic profiles. Anal Chem. (2007) 79:6995–7004. 10.1021/ac0708588 17702530

[5] Ankley GT Daston GP Degitz SJ Denslow ND Hoke RA Kennedy SW. Toxicogenomics in regulatory ecotoxicology. Environ Sci Technol. (2006) 40:4055–65. 10.1021/es0630184 16856717 PMC1892581

[6] Yan M Xu G. Current and future perspectives of functional metabolomics in disease studies-a review. Anal Chim Acta. (2018) 1037:41–54. 10.1016/j.aca.2018.04.006 30292314

[7] Zhou N Sun YP Zheng X Wang Q Yang Y Bai Z. A metabolomics-based strategy for the mechanism exploration of traditional Chinese medicine: descurainia sophia seeds extract and fractions as a case study. Evid Based Complement Alternat Med. (2017) 2017:2845173. 10.1155/2017/2845173 28932251 PMC5592412

[8] Qu W Chen Z Hu X Zou T Huang Y Zhang Y. Profound perturbation in the metabolome of a canine obesity and metabolic disorder model. Front Endocrinol (Lausanne). (2022) 13:849060. 10.3389/fendo.2022.849060 35620391 PMC9128610

[9] Pann P de Angelis MH Prehn C. Mouse age matters: how age affects the murine plasma metabolome. Metabolites. (2020) 10:472. 10.3390/metabo10110472 33228074 PMC7699431

[10] Cyr A Kohut L Chambers L Stratimirovic S Zuckerbraun B. Circulating Metabolomic analysis following cecal ligation and puncture in young and aged mice reveals age-associated temporal shifts in nicotinamide and histidine/histamine metabolic pathways. Oxid Med Cell Longev. (2021) 2021:5534241. 10.1155/2021/5534241 34512866 PMC8433009

[11] Wu CS Muthyala S Klemashevich C Ufondu U Menon R Chen Z. Age-dependent remodeling of gut microbiome and host serum metabolome in mice. Aging (Albany NY). (2021) 13:6330–45. 10.18632/aging.202525 33612480 PMC7993679

[12] Deda O Gika HG Taitzoglou I Raikos N Theodoridis G. Impact of exercise and aging on rat urine and blood metabolome An LC-MS Based Metabolomics Longitudinal Study. Metabolites. (2017) 7:10. 10.3390/metabo7010010 28241477 PMC5372213

[13] Huang Y Xiao M Ou J Lv Q Wei Q Chen Z. Identification of the urine and serum metabolomics signature of gout. Rheumatology (Oxford). (2020) 59:2960–9. 10.1093/rheumatology/keaa018 32134107

[14] Chiu C-Y Cheng M-L Chiang M-H Wang C-J Tsai M-H Lin G. Metabolomic analysis reveals distinct profiles in the plasma and prine associated with IgE reactions in childhood asthma. J Clin Med. (2020) 9:887. 10.3390/jcm9030887 32213896 PMC7141511

[15] Gao J Zhou N Wu Y Lu M Wang Q Xia C. Urinary metabolomic changes and microbiotic alterations in presenilin1/2 conditional double knockout mice. J Transl Med. (2021) 19:351. 10.1186/s12967-021-03032-9 34399766 PMC8365912

[16] Chiu CY Yeh KW Lin G. Metabolomics reveals dynamic metabolic changes associated with age in early childhood. PLoS ONE. (2016) 11:e0149823. 10.1371/journal.pone.0149823 26914934 PMC4767415

[17] Darst BF Koscik RL Hogan KJ. Longitudinal plasma metabolomics of aging and sex. Aging (Albany NY). (2019) 11:1262–82. 10.18632/aging.101837 30799310 PMC6402508

[18] Chen L Zhang J Teh JPY Cheon BK Yang Y Schlundt J. Comparative blood and urine metabolomics analysis of healthy elderly and young male Singaporeans. J Proteome Res. (2020) 19:3264–75. 10.1021/acs.jproteome.0c00215 32434331

[19] D'Ascenzo N Antonecchia E Angiolillo A. Metabolomics of blood reveals age-dependent pathways in Parkinson's Disease. Cell Biosci. (2022) 12:102. 10.1186/s13578-022-00831-5 35794650 PMC9258166

[20] Banton S Pezzali JG Verbrugghe A. Addition of dietary methionine but not dietary taurine or methyl donors/receivers to a grain-free diet increases postprandial homocysteine concentrations in adult dogs. J Anim Sci. (2021) 99:skab223. 10.1093/jas/skab223 34333630 PMC8420682

[21] Price AK de Godoy MRC Harper TA. Effects of dietary calcium fructoborate supplementation on joint comfort and flexibility and serum inflammatory markers in dogs with osteoarthritis. J Anim Sci. (2017) 95:2907–16. 10.2527/jas2017.1588 28727103

[22] Nicholatos JW Robinette TM Tata SVP. Cellular energetics and mitochondrial uncoupling in canine aging. Geroscience. (2019) 41:229–42. 10.1007/s11357-019-00062-6 30937823 PMC6544733

[23] Kaeberlein M Creevy KE Promislow DE. The dog aging project: translational geroscience in companion animals. Mamm Genome. (2016) 27:279–88. 10.1007/s00335-016-9638-7 27143112 PMC4936929

[24] Harper EJ Hackett R Wilkinson J Heaton P. Age-related variations in hematologic and plasma biochemical test results in Beagles and Labrador Retrievers. J Am Vet Med Assoc. (2003) 223:1436–42. 10.2460/javma.2003.223.1436 14627092

[25] Nakayama S Koie H Kanayama K Katakai Y Ito-Fujishiro Y Sankai T. Establishment of reference values for complete blood count and blood gases in cynomolgus monkeys (Macaca fascicularis). J Vet Med Sci. (2017) 79:881–8. 10.1292/jvms.16-0638 28381665 PMC5447977

[26] Brenten T Morris PJ. Salt Age-associated C, and breed-associated variations in haematological and biochemical variables in young labrador retriever and miniature schnauzer dogs. Vet Rec Open. (2016) 3(1): e000166. 10.1136/vetreco-2015-000166 27252875 PMC4879334

[27] Ishii T Hori H Ishigami M. Background data for hematological and blood chemical examinations in juvenile beagles. Exp Anim. (2013) 62:1–7. 10.1538/expanim.62.1 23357940

[28] Rørtveit R Saevik BK Eggertsdóttir AV. Age-related changes in hematologic and serum biochemical variables in dogs aged 16-60 days. Vet Clin Pathol. (2015) 44:47–57. 10.1111/vcp.12220 25559636

[29] Shiel RE Brennan SF O'Rourke LG. Hematologic values in young pretraining healthy Greyhounds. Vet Clin Pathol. (2007) 36:274–7. 10.1111/j.1939-165X.2007.tb00223.x 17806076

[30] Goolsby MJ. Screening, diagnosis, and monitoring of hepatic injury. J Am Acad Nurse Pract. (2003) 15:434–7. 10.1111/j.1745-7599.2003.tb00328.x 14606131

[31] Han X Bao X Lou Q Xie X Zhang M Zhou S. Nicotinamide riboside exerts protective effect against aging-induced NAFLD-like hepatic dysfunction in mice. PeerJ. (2019) 7:e7568. 10.7717/peerj.7568 31523515 PMC6717504

[32] Heck SO Fulco BCW Quines CB Oliveira CES Leite MR Cechella JL. Combined therapy with swimming exercise and a diet supplemented with diphenyl diselenide is effective against age-related changes in the hepatic metabolism of rats. J Cell Biochem. (2017) 118:1574–82. 10.1002/jcb.25819 27918086

[33] Sun H Liu Q Wang X Li M Fan Y Song G. The longitudinal increments of serum alanine aminotransferase increased the incidence risk of metabolic syndrome: a large cohort population in China. Clin Chim Acta. (2019) 488:242–7. 10.1016/j.cca.2018.10.033 30381232

[34] Vimalraj S. Alkaline phosphatase: Structure, expression and its function in bone mineralization. Gene. (2020) 754:144855. 10.1016/j.gene.2020.144855 32522695

[35] Jo S Han J Lee YL Yoon S Lee J Wang SE. Regulation of osteoblasts by alkaline phosphatase in ankylosing spondylitis. Int J Rheum Dis. (2019) 22:252–61. 10.1111/1756-185X.13419 30415492

[36] Huergo LF Dixon R. The emergence of 2-oxoglutarate as a master regulator metabolite. Microbiol Mol Biol Rev. (2015) 79:419–35. 10.1128/MMBR.00038-15 26424716 PMC4651028

[37] He L Li H Huang N. Effects of alpha-ketoglutarate on glutamine metabolism in piglet enterocytes in *vivo* and in *vitro*. J Agric Food Chem. (2016) 64:2668–73. 10.1021/acs.jafc.6b00433 27018713

[38] Jiang Q Adebowale TO Tian J. Effects of maternal alpha-ketoglutarate supplementation during lactation on the performance of lactating sows and suckling piglets. Arch Anim Nutr. (2019) 73:457–71. 10.1080/1745039X.2019.1640023 31454268

[39] Yuan H Cheung CY Hilbers PA. Flux balance analysis of plant metabolism: the effect of biomass composition and model Structure on model predictions. Front Plant Sci. (2016) 7:537. 10.3389/fpls.2016.00537 27200014 PMC4845513

[40] Niciu MJ Kelmendi B Sanacora G. Overview of glutamatergic neurotransmission in the nervous system. Pharmacol Biochem Behav. (2012) 100:656–64. 10.1016/j.pbb.2011.08.008 21889952 PMC3253893

[41] Young SZ Bordey A. GABA's control of stem and cancer cell proliferation in adult neural and peripheral niches. Physiology (Bethesda). (2009) 24:171–85. 10.1152/physiol.00002.2009 19509127 PMC2931807

[42] Roalf DR Sydnor VJ Woods M A. quantitative meta-analysis of brain glutamate metabolites in aging. Neurobiol Aging. (2020) 95:240–9. 10.1016/j.neurobiolaging.2020.07.015 32866885 PMC7609608

[43] Mussai F Egan S Higginbotham-Jones J. Arginine dependence of acute myeloid leukemia blast proliferation: a novel therapeutic target. Blood. (2015) 125:2386–96. 10.1182/blood-2014-09-600643 25710880 PMC4416943

[44] Marini JC Agarwal U Robinson JL Yuan Y Didelija IC Stoll B. The intestinal-renal axis for arginine synthesis is present and functional in the neonatal pig. Am J Physiol Endocrinol Metab. (2017) 313:E233–42. 10.1152/ajpendo.00055.2017 28611027 PMC5582884

[45] Bol S Bunnik EM. Lysine supplementation is not effective for the prevention or treatment of feline herpesvirus 1 infection in cats: a systematic review. BMC Vet Res. (2015) 11:284. 10.1186/s12917-015-0594-3 26573523 PMC4647294

[46] Rashid J Kumar SS Job KM. Therapeutic potential of citrulline as an arginine supplement: a clinical pharmacology review. Paediatr Drugs. (2020) 22:279–93. 10.1007/s40272-020-00384-5 32140997 PMC7274894

[47] Nagaya N Uematsu M Oya H. Short-term oral administration of L-arginine improves hemodynamics and exercise capacity in patients with precapillary pulmonary hypertension. Am J Respir Crit Care Med. (2001) 163:887–91. 10.1164/ajrccm.163.4.2007116 11282761

[48] Sharif Kashani B Tahmaseb Pour P Malekmohammad M. Oral l-citrulline malate in patients with idiopathic pulmonary arterial hypertension and Eisenmenger Syndrome: a clinical trial. J Cardiol. (2014) 64:231–5. 10.1016/j.jjcc.2014.01.003 24525046

[49] El-Hattab AW Emrick LT Hsu JW Chanprasert S. Impaired nitric oxide production in children with MELAS syndrome and the effect of arginine and citrulline supplementation. Mol Genet Metab. (2016) 117:407–12. 10.1016/j.ymgme.2016.01.010 26851065 PMC4818739

[50] Tan B Li XG Kong X. Dietary L-arginine supplementation enhances the immune status in early-weaned piglets. Amino Acids. (2009) 37:323–31. 10.1007/s00726-008-0155-1 18712273

[51] Yun W Song M Lee J. Arginine addition in a diet for weaning pigs can improve the growth performance under heat stress. J Anim Sci Technol. (2020) 62:460–7. 10.5187/jast.2020.62.4.460 32803178 PMC7416154

[52] Visek WJ. An update of concepts of essential amino acids. Annu Rev Nutr. (1984) 4:137–55. 10.1146/annurev.nu.04.070184.001033 6380536

[53] Kang M Yin J Ma J. Effects of dietary histidine on growth performance, serum amino acids, and intestinal morphology and microbiota communities in low protein diet-fed piglets. Mediators Inflamm. (2020) 2020:1240152. 10.1155/2020/1240152 33354159 PMC7735825

[54] Lim JK Narang PK Overman DO. Beneficial effects of methionine and histidine in aspirin solutions on gastric mucosal damage in rats. J Pharm Sci. (1979) 68:295–8. 10.1002/jps.2600680310 423116

[55] Moradi-Arzeloo M Farshid AA Tamaddonfard E Asri-Rezaei S. Effects of histidine and vitamin C on isoproterenol-induced acute myocardial infarction in rats. Vet Res Forum. (2016) 7:47–54. 27226887 PMC4867037

[56] Gibbs NK. l-Histidine Supplementation in adults and young children with atopic dermatitis (eczema). J Nutr. (2020) 150:2576S−9S. 10.1093/jn/nxaa200 33000160

[57] Mahootchi E Cannon Homaei S Kleppe R. GADL1 is a multifunctional decarboxylase with tissue-specific roles in β-alanine and carnosine production. Sci Adv. (2020) 6:eabb3713. 10.1126/sciadv.abb3713 32733999 PMC7367687

[58] Baguet A Everaert I Achten E. The influence of sex, age and heritability on human skeletal muscle carnosine content. Amino Acids. (2012) 43:13–20. 10.1007/s00726-011-1197-3 22170500

[59] Sivamaruthi BS Fern LA Ismail DSNRPH Chaiyasut C. The influence of probiotics on bile acids in diseases and aging. Biomed Pharmacother. (2020) 128:110310. 10.1016/j.biopha.2020.110310 32504921

[60] Kim YK Kwon OS Her KH. The grade of nonalcoholic fatty liver disease is an independent risk factor for gallstone disease: an observational study. Medicine (Baltimore). (2019) 98:e16018. 10.1097/MD.0000000000016018 31277096 PMC6635222

[61] Han Q Li H Jia M Wang L Zhao Y Zhang M. Age-related changes in metabolites in young donor livers and old recipient sera after liver transplantation from young to old rats. Aging Cell. (2021) 20:e13425. 10.1111/acel.13425 34157207 PMC8282239

[62] Kroll J. Bile acids, chaperones, mammalian longevity. Rejuvenation Res. (2012) 15:210–2. 10.1089/rej.2011.1286 22533434

[63] Hoefgen R Nikiforova VJ. Metabolomics integrated with transcriptomics: assessing systems response to sulfur-deficiency stress. Physiol Plant. (2008) 132:190–8. 10.1111/j.1399-3054.2007.01012.x 18251860

[64] Wood ZA Schröder E Harris J Poole L. Structure, mechanism and regulation of peroxiredoxins. Trends Biochem Sci. (2003) 28:32–40. 10.1016/S0968-0004(02)00003-8 12517450

[65] Liao D Pajak A Karcz SR Chapman BP Sharpe AG Austin RS. Transcripts of sulphur metabolic genes are co-ordinately regulated in developing seeds of common bean lacking phaseolin and major lectins. J Exp Bot. (2012) 63:6283–95. 10.1093/jxb/ers280 23066144 PMC3481216

[66] Herzenberg LA De Rosa SC Dubs JG Roederer M Anderson MT Ela SW. Glutathione deficiency is associated with impaired survival in HIV disease. Proc Natl Acad Sci U S A. (1997) 94:1967–72. 10.1073/pnas.94.5.1967 9050888 PMC20026

[67] Hildebrandt W Alexander S Bärtsch P Dröge W. Effect of N-acetyl-cysteine on the hypoxic ventilatory response and erythropoietin production: linkage between plasma thiol redox state and O(2) chemosensitivity. Blood. (2002) 99:1552–5. 10.1182/blood.V99.5.1552 11861267

[68] Droge W. Oxidative stress and ageing: is ageing a cysteine deficiency syndrome? Philos Trans R Soc Lond B Biol Sci. (2005) 360:2355–72. 10.1098/rstb.2005.1770 16321806 PMC1569588

[69] Satyaraj E Reynolds A Pelker R Labuda J Zhang P Sun P. Supplementation of diets with bovine colostrum influences immune function in dogs. Br J Nutr. (2013) 110:2216–21. 10.1017/S000711451300175X 23773360

