# Supplementary material for: Comparative plasma and urine metabolomics analysis of juvenile and adult canines
Source: Front Vet Sci. 2023 Jan 9;9:1037327. doi: 10.3389/fvets.2022.1037327 (PMC9868312; doi:10.3389/fvets.2022.1037327)
Supplement: Supplementary file 1 [file Data_Sheet_1.PDF]

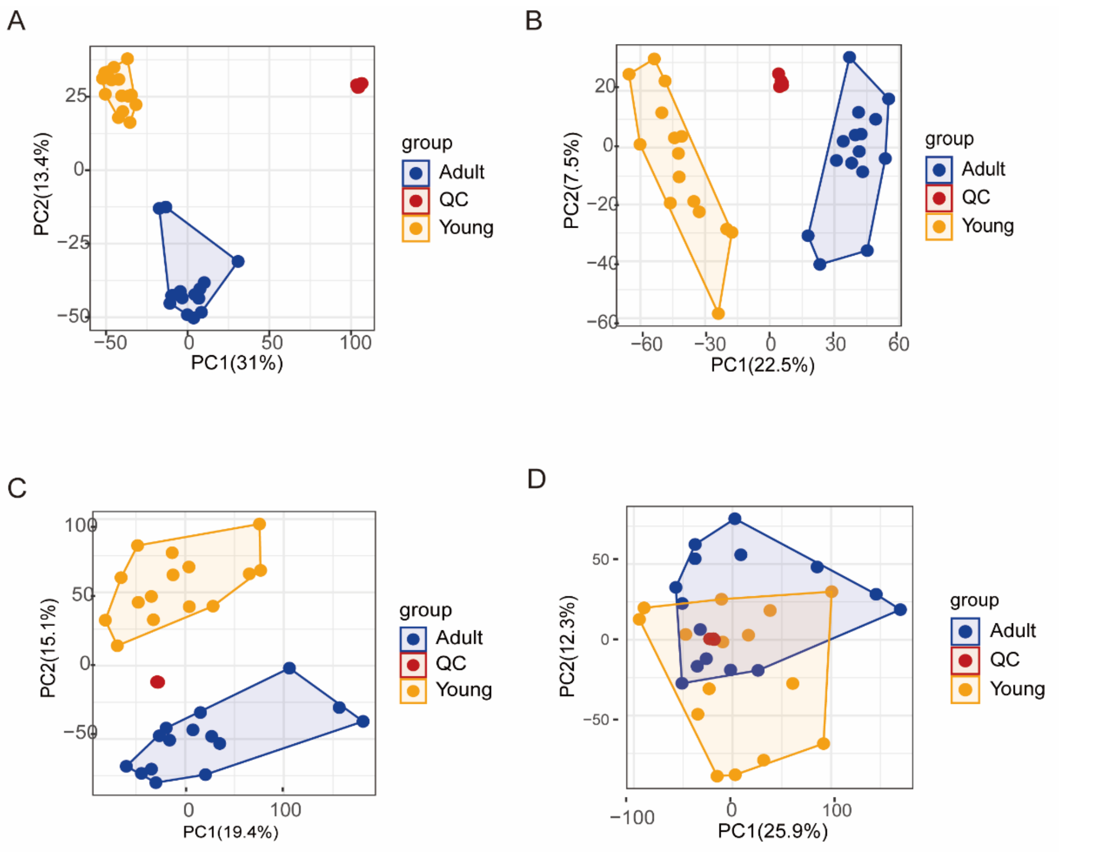

The PCA analysis of all samples including quality control (QC) samples is shown in Supplementary Figure S1. (A) PCA analysis in positive ion mode (plasma metabolome); (B) PCA analysis in negative ion mode (plasma metabolome); (C) PCA analysis in positive ion mode (urine metabolome); (D) PCA analysis in negative ion mode (urine metabolome);
